# Investigating feasibility of 2021 WHO protocol for cervical cancer screening in underscreened populations: PREvention and SCReening Innovation Project Toward Elimination of Cervical Cancer (PRESCRIP-TEC)

**DOI:** 10.1186/s12889-022-13488-z

**Published:** 2022-07-15

**Authors:** Marat Sultanov, Janine de Zeeuw, Jaap Koot, Jurjen van der Schans, Jogchum J. Beltman, Marlieke de Fouw, Marek Majdan, Martin Rusnak, Naheed Nazrul, Aminur Rahman, Carolyn Nakisige, Arathi P. Rao, Keerthana Prasad, Shyamala Guruvare, Regien Biesma, Marco Versluis, Geertruida H. de Bock, Jelle Stekelenburg

**Affiliations:** 1 Department of Health Sciences, Global Health Unit, University Medical Center Groningen, University of Groningen, Groningen, Netherlands; 2 Department of Economics, Econometrics and Finance, Faculty of Economics and Business, University of Groningen, Groningen, Netherlands; 3 Department of Gynecology, Leiden University Medical Centre, Leiden University, Leiden, Netherlands; 4 Institute for Global Health and Epidemiology, Department of Public Health, Faculty of Health Sciences and Social Work, Trnava University, Trnava, Slovak Republic; 5 Friendship Foundation, Dhaka, Bangladesh; 6 Health System and Population Studies Division, icddr,b, Dhaka, Bangladesh; 7 Uganda Cancer Institute, Kampala, Uganda; 8 Prasanna School of Public Health, Manipal Academy of Higher Education, Manipal, India; 9 Manipal School of Information Sciences, Manipal Academy of Higher Education, Manipal, India; 10 Kasturba Medical College, Manipal Academy of Higher Education, Manipal, India; 11 Department of Obstetrics and Gynecology, University Medical Center Groningen, University of Groningen, Groningen, Netherlands; 12 Department of Epidemiology, University Medical Center Groningen, University of Groningen, Groningen, Netherlands; 13 Department of Obstetrics and Gynecology, Medical Center Leeuwarden, Leeuwarden, Netherlands

**Keywords:** Cervical cancer, Cervical cancer screening, Human papillomavirus testing, Implementation, Bangladesh, India, Slovakia, Uganda

## Abstract

**Background:**

High-risk human papillomavirus (hrHPV) testing has been recommended by the World Health Organization as the primary screening test in cervical screening programs. The option of self-sampling for this screening method can potentially increase women’s participation. Designing screening programs to implement this method among underscreened populations will require contextualized evidence.

**Methods:**

PREvention and SCReening Innovation Project Toward Elimination of Cervical Cancer (PRESCRIP-TEC) will use a multi-method approach to investigate the feasibility of implementing a cervical cancer screening strategy with hrHPV self-testing as the primary screening test in Bangladesh, India, Slovak Republic and Uganda. The primary outcomes of study include uptake and coverage of the screening program and adherence to follow-up. These outcomes will be evaluated through a pre-post quasi-experimental study design. Secondary objectives of the study include the analysis of client-related factors and health system factors related to cervical cancer screening, a validation study of an artificial intelligence decision support system and an economic evaluation of the screening strategy.

**Discussion:**

PRESCRIP-TEC aims to provide evidence regarding hrHPV self-testing and the World Health Organization’s recommendations for cervical cancer screening in a variety of settings, targeting vulnerable groups. The main quantitative findings of the project related to the impact on uptake and coverage of screening will be complemented by qualitative analyses of various determinants of successful implementation of screening. The study will also provide decision-makers with insights into economic aspects of implementing hrHPV self-testing, as well as evaluate the feasibility of using artificial intelligence for task-shifting in visual inspection with acetic acid.

**Trial registration:**

ClinicalTrials.gov, NCT05234112. Registered 10 February 2022

**Supplementary Information:**

The online version contains supplementary material available at (10.1186/s12889-022-13488-z).

## Background

Despite being largely preventable through human papillomavirus (HPV) vaccination and screening programs, cervical cancer remains a global health challenge. With estimated 604,000 new cases and 342,000 deaths worldwide in 2020, cervical cancer is the fourth most frequently diagnosed cancer and the fourth leading cause of death from cancer among women [1]. Significant reductions in the burden of cervical cancer have occurred in high-income countries (HICs) in recent decades. However, vulnerable groups in HICs continue to be affected disproportionately, and the vast majority of cases and deaths occur in low- and middle-income countries (LMICs) [2].

Screening methods for cervical cancer include cytology-based methods, visual inspection methods and high-risk human papillomavirus (hrHPV) testing. Cytology-based cervical cancer screening programs (using conventional Pap smear or liquid-based cytology) have led to major reductions in cervical cancer burden in HICs. However, this method of screening has not been implemented in many resource-poor settings due to reasons such as lack of infrastructure and limited healthcare workforce capacity [3]. As a result, visual inspection methods, in particular visual inspection with acetic acid (VIA), have been introduced in many LMICs instead. Nevertheless, both the sensitivity and specificity of visual inspection methods are variable and dependent on the healthcare provider, which can result in variation in the quality of screening services, particularly in resource-poor settings [4].

Infection with high-risk HPV (hrHPV) types is established as the main risk factor for the development of cervical cancer [5]. HrHPV testing offers higher sensitivity compared to VIA and cytology [6], which allows for longer intervals between repeated screenings [7]. Moreover, compared to provider-collected approach and to other types of screening, the option of self-sampling for hrHPV testing has the potential to increase uptake of cervical cancer screening through reducing socioeconomic, cultural and logistical barriers to participation in screening [8]. Therefore, HPV self-testing as the primary screening method could be a more cost-effective and affordable option compared to other screening methods [9]. The World Health Organization’s (WHO) guidelines for cervical cancer prevention, updated in 2021, recommend HPV testing (either self-sampled or provider-collected) as the primary screening test, either in a screen-and-treat approach, whereby all women identified as positive are offered ablative or excisional treatment, or a screen, triage and treat approach [10]. In the latter approach, VIA and cytology-based methods are recommended as options for triage following a positive hrHPV test to identify women eligible for treatment of precancerous lesions. The choice of triage method in each country ultimately depends on feasibility and resource availability.

In the screen, triage and treat approach, LMICs with established VIA screening capacity can choose to use VIA for triage and introduce HPV testing as the primary screening method. However, in this approach, quality of VIA screening can be inconsistent due to a number of factors, such as experience and skill level of the healthcare provider performing the test, and the source of light for visualization [11, 12]. The variability in VIA accuracy resulting from these factors can potentially be reduced by artificial intelligence (AI) algorithms. AI methods could provide decision support for lower-level healthcare workers performing VIA, which could be particularly relevant for LMICs with limited healthcare workforce capacities.

It has been estimated that a combination of scaled-up cervical cancer screening and HPV vaccination will be required to achieve cervical cancer elimination targets throughout the next century [13]. The WHO Global Strategy to eliminate cervical cancer, introduced in 2020, includes a global target of 70% for screening coverage [14]. Achieving sufficient coverage and uptake of screening, particularly in LMICs and among vulnerable groups in HICs, is seen as crucial for eliminating cervical cancer in the global context. While the efficacy of the recommended screening approaches has long been established, the multidimensional nature of cervical cancer prevention will require situating the various potential barriers and facilitators associated with these services in the individual countries’ context in order to inform national and global efforts supporting the WHO elimination strategy.

The issues presented above, along with the lack of contextual, country-specific knowledge to support the implementation of the WHO protocol to reach underscreened populations, motivate the conception of the PREvention and SCReening Innovation Project Toward Elimination of Cervical Cancer (PRESCRIP-TEC). This paper describes the design of PRESCRIP-TEC, which aims to study the feasibility of implementing the latest WHO recommendations for cervical cancer screening in addition to existing community-based cervical cancer screening programs.

## Methods

### Primary and secondary objectives

The primary objective of PRESCRIP-TEC is to evaluate the changes in coverage and uptake of primary screening through hrHPV self-testing, and adherence to follow-up and treatment recommendations after screening, resulting from an enhanced screening program introduced in Bangladesh, India, Uganda and the Slovak Republic, and compared to the current screening program in each of the countries. Secondary objectives include analyzing the barriers and facilitators related to the uptake of hrHPV self-sampling (client-related and health system-related factors), performing a validation study of an AI decision support system (AI-DSS) for VIA, and conducting a model-based economic evaluation of the potential cost-effectiveness of the enhanced screening program for each of the countries.

### Study setting

The project will be conducted in four countries: three LMICs (Bangladesh, India and Uganda) and one HIC (Slovak Republic) [15]. These countries have been selected because of variations concerning high cervical cancer incidence and/or low uptake of screening, different implementation settings and hard-to-reach areas and vulnerable population groups. In addition, these countries have been early adopters of screening and treatment methods in the past. The information regarding the demographic and cervical cancer profiles of the countries is summarized in Table 1. In each country, study areas and vulnerable populations were selected based on context analysis.

**Table 1:** Summary of country settings

	Bangladesh	India	Slovak Republic	Uganda
Population (million) [16]	164.69	1380	5.46	45.74
GDP per capita, PPP (current international $) [16]	5136.7	6501.5	32014.6	2293.5
Cervical cancer incidence rate (crude, per 100 000) [17]	10.2	18.7	24.9	30
Cervical cancer incidence rate (age-standardized, per 100 000) [17]	10.6	18.0	16.6	56.2
Cervical cancer mortality (crude, per 100 000) [17]	6.1	11.7	10.1	19.9
Cervical cancer mortality (age-standardized, per 100 000) [17]	6.7	11.4	5.3	41.4
Available/recommended method of screening	VIA	VIA/Pap smear	Pap smear	VIA
Type of screening	Opportunistic	Opportunistic	Opportunistic	Opportunistic
Target age group in the project (years)	30-60	35-63	19-64	30-49

### Participants

The enhanced screening program will target women in age groups defined in accordance with the existing screening policy of the countries. In Bangladesh, the screening intervention will target women aged 30-60, living in several remote areas. In the northern part of Bangladesh, four districts (Kurigram, Gaibandha, Bogura and Sirajganj) were selected for the study and one district (Sathkhira) was selected in the southern part of the country. In India, women aged 35-63 will be targeted in rural and urban areas, including urban slum and hilly regions, in Udupi, Sikkim, Kolkata, Bangalore and Mumbai. In addition, HIV positive women, sex workers and other vulnerable groups will be approached in Bangalore by participating institutions. In the Slovak Republic, the target groups are women aged 19-64 from Roma communities in 16 districts across Prešov, Banská Bystrica and Košice regions in the eastern part of the country and women aged 19-64 working in 15 car factories across all regions. In Uganda, women aged 30-49 living in rural areas in the Kakumiro district will be targeted.

Eligible women who can be offered the hrHPV self-test in the target age group and regions are defined as follows:

- They are non-pregnant women eligible for participation in the screening program according to the national screening policy;
- Prior to the study, they have not been screened within the interval defined by the national screening policy.

To participate in the research component (primary and secondary research objectives), the following criteria will be applied to women eligible for the hrHPV self-test as described above, as well as other respondents, including husbands, household decision makers and other stakeholders:

- They understand information about the study and provide informed consent for participation in the study;
- They have the verbal skills to respond to questions and/or engage in an interview.

### Intervention

Prior to the introduction of the screening program, information campaigns will be conducted to reach the target audience. These will include, among others, communicating through radio, social media, phone messaging, theater, flyers, posters and other awareness-raising activities. The campaign formats will be chosen based on the country context. We aim to reach over 100,000 men and women per country through online and offline communication channels.

The project aims to provide screening services for 6000-8000 eligible women in each country. The project will apply a community-based approach, in which eligible women will be visited in their homes and/or mobilized through outreach efforts and will be offered a self-test for hrHPV screening. Depending on the women’s preferences and the community circumstances, the self-test can be taken at their homes or at the nearby clinics with the possibility of assistance by a healthcare worker.

**Fig. 1:**
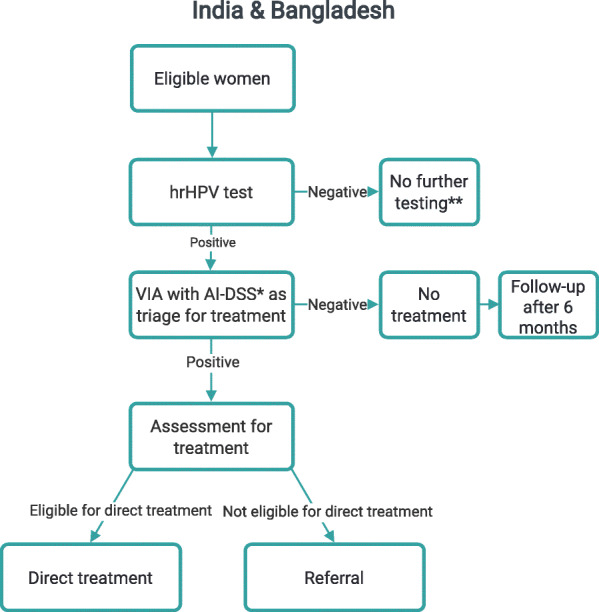
Screening strategy - India and Bangladesh. * - AI-DSS in study mode only, treatment decisions will be based on manual VIA ** - Implies only absence of further testing within the project, national guidelines for screening intervals apply

**Fig. 2:**
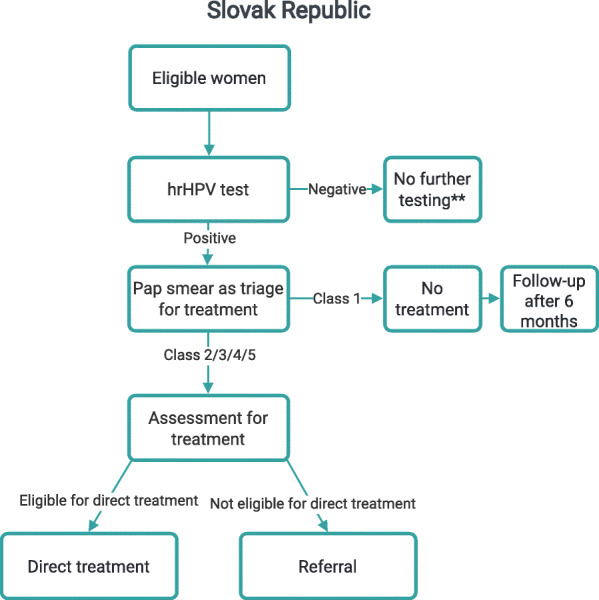
Screening strategy - Slovak Republic. * - AI-DSS in study mode only, treatment decisions will be based on manual VIA ** - Implies only absence of further testing within the project, national guidelines for screening intervals apply

**Fig. 3:**
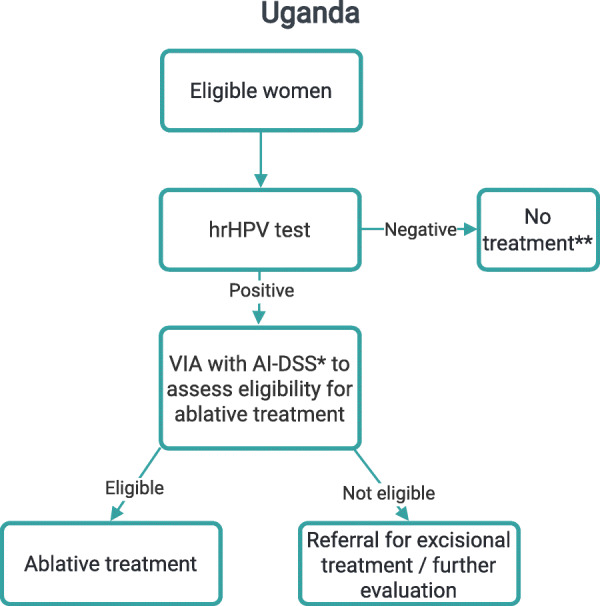
Screening strategy - Uganda. * - AI-DSS in study mode only, treatment decisions will be based on manual VIA ** - Implies only absence of further testing within the project, national guidelines for screening intervals apply

The screening strategies implemented in the countries as part of PRESCRIP-TEC are presented in Figs. 1, 2, 3. In Bangladesh, India and Slovak Republic, in case of a positive hrHPV result, the women will be invited for the follow-up examination in a screen, triage and treat approach. This follow-up examination will involve VIA, aided by AI-DSS, in Bangladesh and India, while in Slovak Republic this examination will involve the use of Pap smear (without AI-DSS). This difference is due to current screening policies in the participating countries, upon which PRESCRIP-TEC builds. In Uganda, the screen-and-treat approach will be used, in which all women identified as hrHPV-positive will be treated in accordance with the national guideline. Hereby, AI-DSS will be used in visual assessment of women for eligibility for thermal ablation treatment. Alternatively, depending on availability, cryotherapy may be used. The clinical performance of the AI-DSS for VIA will be evaluated in this study as a secondary objective.

In Bangladesh, India and Uganda, direct treatment during the same follow-up visit will be provided if lesions are identified as eligible. Otherwise, women will be referred for treatment in accordance with the existing national guidelines.

Subsequent diagnosis and treatment are outside the scope of evaluation in this project. In case of negative hrHPV test, women will be advised to follow the national guidelines regarding screening intervals for their next screening test.

### Outcomes

The main outcomes linked to the primary objective of PRESCRIP-TEC are described in Table 2.

**Table 2:** Outcomes

Outcome	Definition
Coverage	Proportion of women who hand in the hrHPV swab out of all women eligible for screening who belong to the target group in the research area
Uptake	Proportion of women who hand in the hrHPV swab out of all women who received the self-swab after having been actively approached to participate in screening
Adherence to follow-up	Proportion of women receiving the follow-up examination (VIA or Pap smear) out of all women identified as hrHPV positive

### Logic model

To represent the research components of PRESCRIP-TEC, we adapted the Implementation Research Logic Model [18], refer to the Additional file 1. The determinants are divided into client-related factors, adapted from [19], and health system factors, adapted from [20]. The determinants are linked to the intervention and the implementation strategies, which lead to outcomes through the hypothesized mechanisms.

### Study design

PRESCRIP-TEC is an implementation study, in which a multi-method approach will be applied to evaluate the enhanced screening program in four countries. For the primary objective, the design of the study can be described as a pre-post quasi-experimental study with non-equivalent control groups [21]. Control areas have been selected in each participating country to match the designated intervention areas on the availability of screening services offered and the target population characteristics.

The pre-post comparison to measure the main outcomes will be conducted using the difference-in-differences study design. In this approach, the coverage, uptake and adherence to follow-up will be compared between intervention areas and control areas in each country at baseline (prior to the implementation of the screening intervention) and end-line (after the full implementation) through retrospective surveys. Additional outcomes of the follow-up test and the proportion of women receiving direct treatment after the examination will be analyzed as well. Baseline data in the intervention and control areas will be collected retrospectively at the same time as the endline survey. Where possible, the baseline data will be validated using other sources, such as historical data and data collected as part of the secondary objectives.

#### Sample size

Analysis of data will be performed at the cluster level, which will be identified for each country (clinic, village or district). Sample sizes for varying numbers of potential clusters were estimated using an approach for power calculation for group-randomized trials with several assumptions [22].

Uptake was used as the main outcome for sample size considerations. The absolute increase in uptake was assumed at 20%. Based on the uptake of cervical cancer screening, the intraclass correlation coefficient was assumed to be 0.02. This also ranged between 0.01 and 0.05, depending on the type of cluster. The variance reduction was conservatively assumed to be 1. Type I error (alpha) was set at 5% and type II error (beta) was set at 80%. Based on these assumptions, we expect the sample sizes for the pre-post comparison to vary between 375 and 1500. These final sample sizes are subject to identification of the number and type of clusters in each country.

#### Statistical analysis

To determine if the intervention resulted in significant changes in uptake, coverage and adherence to follow-up in the intervention areas compared to control areas, multivariable regression analysis will be performed.

### Secondary objectives

#### Client-related factors

For the purposes of this study, we categorize potential factors influencing the primary outcomes of uptake, coverage and adherence to follow-up into client-related factors and health system-related factors. Client-related factors can be grouped into contextual, individual or group influences, as well as cancer-specific aspects of screening (Table 3, adapted from [19]).

**Table 3:** Client-related factors

Contextual influences (historic, socio-cultural, environmental, health systems, political factors)	∙ Communication and media environment
	∙ Influential leaders, lobbies
	∙ Historical factors
	∙ Religion, culture
	∙ Gender issues
	∙ Politics
	∙ Geographical barriers
	∙ Perceptions of technology
Individuals and groups	∙ Personal and family experience with cancer
	∙ Beliefs and attitudes regarding screening and prevention
	∙ Knowledge and awareness
	∙ Trust in health system and providers
	∙ Perceived benefits of early treatment
	∙ Social norms in the community
Specific issues related to cervical cancer screening	∙ Attitudes towards gynecological examination
	∙ Attitudes towards privacy or involvement of male providers
	∙ Costs (including indirect costs such as transportation)
	∙ Health systems factors (waiting, returning for screening)

In order to gain insight into community cervical cancer awareness, as well the acceptability, accessibility and adherence to the enhanced screening protocol, a mixed methods approach will be applied and various participants will be enrolled for the study. First, at the community level, men, women and household decision makers will be approached in order to measure baseline community cervical cancer awareness prior to the intervention. The African Woman Awareness of CANcer (AWACAN) tool will be used in order to measure awareness of cervical cancer in baseline and endline household surveys. The AWACAN tool has been developed in order to measure breast and cervical cancer awareness and has shown to be reliable and valid for use in Sub-Saharan Africa [23]. The questionnaire is a mix of open, closed and multiple-choice questions and includes questions about socio-demographic determinants. As PRESCRIP-TEC focuses on cervical cancer, only the questions focusing on cervical cancer awareness were derived from the AWACAN tool. The AWACAN tool contains 41 questions specific to cervical cancer on risk factors, symptoms, lay beliefs, confidence in appraisal, help-seeking behaviors, and 12 questions on barriers to seeking health care for breast and cervical cancer.

After implementation of the intervention, the AWACAN and the Healthcare Systems Trust Framework, [24] will be administered among women in intervention areas and control areas. In addition, among women who accepted the hrHPV self-test a questionnaire of women’s experiences with the test will be administered. The Health Care Systems Trust Framework was developed in India and provides validated questionnaires with 23 items scored on a Likert scale. Each item is part of one of the five domains (perceived quality of services, effective communication, transparency in relations, reliability and technical competence) and has a weight leading to an overall trust score. The questionnaire used to study the acceptability of the hrHPV self-test contains 13 questions related to the self-sampling procedure, as well as knowledge of hrHPV and cervical cancer [25].

Prior to administration of the instruments, country teams familiar with the target population will carefully review the instruments and focus group discussions or individual interviews will be organized in order to contextualize the instruments to ensure compatibility with the target population. In addition, all instruments will be translated into languages of the target populations.

Thereafter, a qualitative follow-up study using in-depth interviews (IDI) and/or focus group discussions (FGD) with eligible women and household decision-makers will be conducted to gain insight into their perceptions of hrHPV self-tests and barriers and facilitators for screening participation. Women will be approached 2-3 weeks after acceptance of hrHPV self-test for an interview or FGD. We aim to conduct 30-40 IDIs and/or FGDs until saturation of themes is achieved. We expect to reach saturation by this number of participants. Thematic content analysis will be used to analyze the transcripts of IDIs/FGDs.

#### Health system factors

The health system factors relevant for cervical cancer screening participation are presented in Table 4 across the “building blocks” of the WHO Health Systems Framework (adapted from [20]). The health system factors will be measured through surveys during the full operation of the enhanced screening program, ongoing assessments during facility visits, and in-depth interviews and/or FGDs with key stakeholders. All facilities in the target areas of the intervention, including mobile units, health posts, health centers or hospitals, will be involved.

**Table 4:** Health system factors

Service delivery	∙ Lack of user-friendly services
	∙ Opportunistic screening instead of proactive screening
	∙ Inadequate privacy or confidentiality
Health workforce	∙ Insufficient or lack of staff (gynecologists, trained nurses or midwives, pathologists, laboratory staff)
	∙ Inadequate staff capabilities
	∙ High turnover of staff
Monitoring and evaluation	∙ Inadequate paper patient files and reporting
	∙ Lack of a reminder system for defaulting patients
Access to medicines / supplies	∙ Unavailability of hrHPV tests;
	∙ Supplies for cryotherapy or thermal ablation only via commercial suppliers
	∙ Insufficient sterilization of equipment
	∙ Lack of maintenance and repair
	∙ Limited number of distributors
Financing	∙ Vertical approach toward community-based programs
	∙ Focus on financing curative care
Governance	∙ Lack of / insufficient implementation of guidelines
	∙ Lack of a functional national screening program

The Facility Based Survey instrument, included in the WHO Cervical Cancer Prevention Toolkit (26), will be used for the survey and ongoing assessment of healthcare facilities in this study objective. This survey instrument is based on the Service Availability and Readiness Assessment tool and is tailored for cervical cancer screening and prevention.

Semi-structured interviews will be conducted among the various key stakeholders on the topics of the progress of implementation, potential moderating factors, and the barriers and facilitators of implementation of the screening protocol. Furthermore, perspectives of healthcare workers may be studied via FGDs. Topic guides for the interviews and focus groups will be developed with the Conceptual Framework for Implementation Fidelity [26] and the Consolidated Framework for Implementation Research [27] serving as frameworks. Follow-up FGDs will be conducted at the end of the project among the same participants to discuss the barriers and facilitators of implementation, and to reflect on the implementation.

#### AI-DSS

An image processing algorithm, developed at the Manipal Academy of Higher Education in India [28], will be evaluated through a validation study in Bangladesh, India and Uganda. This algorithm functions as an Android-based application. The inter-observer variability of the algorithm will be assessed among healthcare workers and gynecological experts. The model performance will be evaluated based on calibration, discrimination, clinical usefulness and error analysis criteria [29].

Before the field implementation of AI-DSS, the diagnostic accuracy of healthcare workers, experts and the AI-DSS will be assessed in a dry run using a database of VIA pictures. Next, the AI device will be used as a “second opinion” for the healthcare workers performing VIA. In case of disagreement between the healthcare worker and the AI-DSS, an expert will provide a second opinion and clinical guidance. Finally, if the AI device is found to demonstrate high convergence either with both the healthcare workers and the experts in the project, or with the experts only (with need for further training for healthcare workers), the study will examine options for task shifting using the AI-DSS.

#### Economic evaluation

To produce relevant and actionable information for the decision-making context in each participating country, a model-based economic evaluation of the screening protocol will be conducted, incorporating a variety of data sources, including the coverage, uptake and adherence to follow-up outcomes, and secondary data. This evaluation will include country-specific cost-effectiveness analyses and budget impact analyses of the screening protocol. The evaluation will compare the screening protocol scenario to a “business-as-usual” scenario of the existing screening policy in each country. The economic analysis aims to inform a business case of implementing the WHO recommendations in LMICs, focusing on the potential affordability of the screening protocol. Special attention will be given to analysis of scenarios for introduction of hrHPV testing in the context of equipment and test costs, as affordability is a major bottleneck for introduction of the new WHO protocol in many countries.

### Data management and monitoring

Data collection will take place in Slovakia, Uganda, Bangladesh, and India under responsibility of local partners in the PRESCRIP-TEC research consortium. Digital data, both raw and processed/analyzed data, as well as the codebooks with metadata, documentation of steps taken or decisions made, scripts and software used for analysis, will be stored at servers in the countries where the data is collected. Data will be pseudonymized before it can be shared to a dedicated server at University Medical Center Groningen.

### Risks

Due to the COVID-19 pandemic, delays in the project could potentially arise as a result of care provision disruptions or COVID-related measures in the selected countries. We anticipate that the project will not experience significant delays once the screening program is introduced by the country teams.

### Safety

Since PRESCRIP-TEC aims to define conditions to scale-up already proven screening strategies, no specific safety risks are expected beyond those identified in the WHO recommendations [10].

## Discussion

PRESCRIP-TEC aims to evaluate the feasibility of hrHPV self-sampling, included in the WHO guidelines, as the primary screening method in the specific contexts of LMICs and vulnerable populations in HICs. The advantages of hrHPV testing compared to other primary screening methods for cervical cancer are well-established [6]. However, hrHPV self-sampling is included in national recommendations only in 17 countries, which constitutes a third of all countries adopting HPV testing as the primary cervical cancer screening method [30], and is often used primarily to reach under-screened populations. The existing evidence for the improved uptake of screening as a result of self-sampling appears to be highly heterogeneous and comes mostly from high-income countries [8]. Moreover, cost-effectiveness literature on the HPV self-sampling in LMICs is lacking, with a particular need for modeling studies of implementation scenarios of self-testing strategies [31]. PRESCRIP-TEC will provide new evidence regarding HPV self-testing and the WHO recommendations in a variety of challenging settings, as well as provide specific recommendations regarding the cost-effectiveness and budget impact of potential screening policies in participating countries in the context of the WHO’s elimination strategy.

Since no strategies can be expected to work universally for all settings and populations, implementation research conducted across diverse settings and population groups can help better assess the sustainability of interventions and the determinants of implementation success [32]. The multi-country approach of PRESCRIP-TEC will build on diverse national screening approaches and help produce more generalizable outputs, compared to a single-country implementation study design. Moreover, as the role of specific client-related and health system-related factors for participation in cervical cancer screening programs will also be analyzed, the project may provide suggestions for targeted action to improve uptake in specific country context.

The COVID-19 pandemic has resulted in disruptions to cancer prevention and treatment services worldwide. More than 50% of countries surveyed by WHO reported postponing screening programs [33], which could result in increased numbers of women overdue for primary cervical cancer screening. Potential trends in relation to these services in LMICs may include further prioritization of communicable diseases over noncommunicable diseases and the acceleration of health inequalities [34]. Moreover, the fact that the production of hrHPV tests and COVID-19 tests uses overlapping reagents and consumables could impair the availability of hrHPV tests. However, the infrastructure and the resources developed for COVID-19 testing, in conjunction with the expansion of manufacturing capacities and investments in diagnostic platforms, could present an opportunity for introduction and expansion of hrHPV testing in the future [35]. In this context, our project will assess the introduction of hrHPV self-testing in resource-constrained settings significantly disrupted by the COVID-19 pandemic. Assessing the implementation of screening strategies in the context of a global pandemic may help inform surveillance and preparedness efforts for future pandemic scenarios.

### Limitations

Due to practical and ethical concerns, quasi-experimental designs are often employed in implementation research. The non-randomized design of this study presents a threat to its internal validity. While we aimed to select the most appropriate control areas/communities for our study to minimize this threat, it is not possible to avoid temporal bias and selection bias inherent to this study design.

While treatment for precancerous lesions will be offered in PRESCRIP-TEC in accordance with the existing treatment strategies in each country, the evaluation of subsequent treatment outcomes is not the focus of our study.

## Supplementary Information

**Additional file 1** Implementation Research Logic Model (adapted).

## Data Availability

Data will be made accessible according to the Open Data Policy of the European Commission Directorate research and Innovation under H2020 financing conditions. PRESCRIP-TEC will apply the guidelines of the Creative Commons licenses. In principle the Attribution-Non-Commercial International license applies (CC BY-NC 4.0).

## Abbreviations

AI: artificial intelligence
AI-DSS: artificial intelligence decision support system
AWACAN: African Woman Awareness of CANcer
FGD: focus group discussion
HIC: high-income country
HPV: human papillomavirus
hrHPV: high-risk human papillomavirus
IDI: in-depth interview
LMIC: low- or middle-income country
PRESCRIP-TEC: PREvention and SCReening Innovation Project Toward Elimination of Cervical Cancer
VIA: visual inspection with acetic acid
WHO: World Health Organization
