# Supplementary figures and images for: Investigating feasibility of 2021 WHO protocol for cervical cancer screening in underscreened populations: PREvention and SCReening Innovation Project Toward Elimination of Cervical Cancer (PRESCRIP-TEC)
Source: BMC Public Health. 2022 Jul 15;22:1356. doi: 10.1186/s12889-022-13488-z (PMC9284962; doi:10.1186/s12889-022-13488-z)

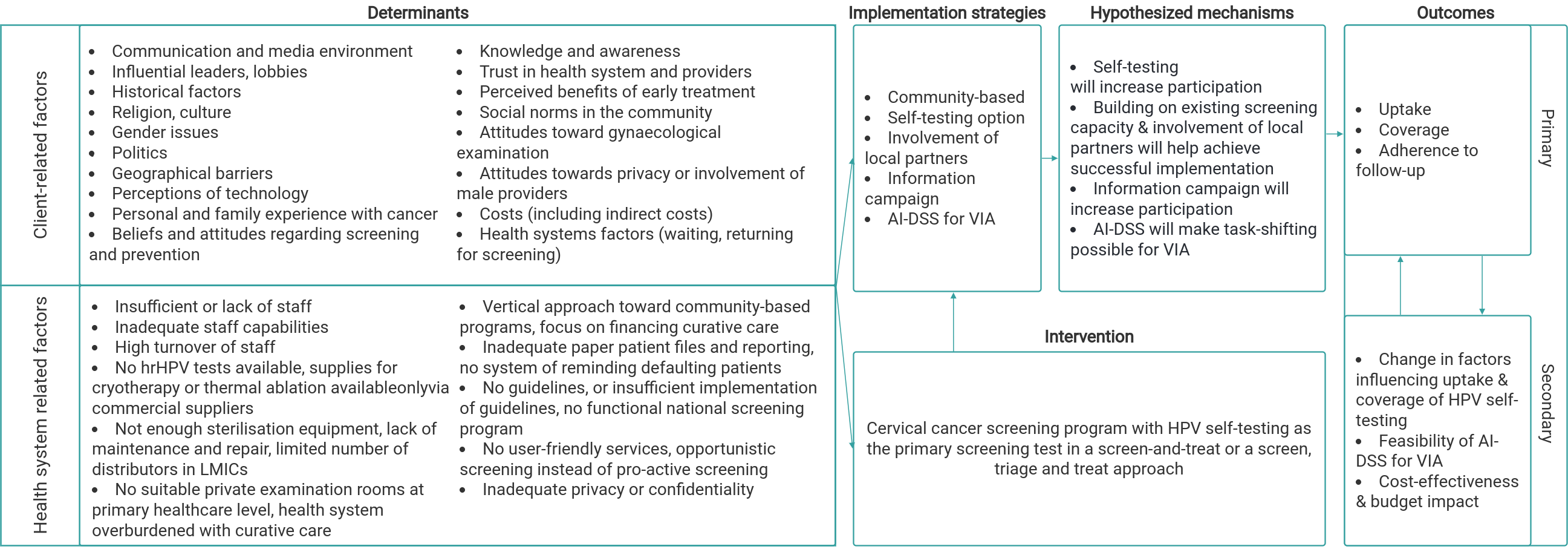

Supplement: Supplementary file 1 — Additional file 1 Implementation Research Logic Model (adapted). [file 12889_2022_13488_MOESM1_ESM.png]
